# Knee Armor Training Program: An 8-Week Sensorimotor Approach to Reducing Knee Injury Risk in Women’s Rugby

**DOI:** 10.3390/jcm14113779

**Published:** 2025-05-28

**Authors:** Karol Jaskulski, Patrycja Bobowik, Bartosz Wysoczański, Agnė Predkelienė, Michał Starczewski

**Affiliations:** 1 Faculty of Rehabilitation, Józef Piłsudski University of Physical Education in Warsaw, Warsaw Marymoncka Str., No. 34, 00-968 Warsaw, Poland; 2 Institute of Educational Research, Vytautas Magnus University, 44248 Kaunas, Lithuania; 3 Education Academy, Vytautas Magnus University, 44248 Kaunas, Lithuania

**Keywords:** injury, anterior cruciate ligament, reactive strength index, dynamic knee valgus, rugby

## Abstract

**Background:** Anterior cruciate ligament (ACL) rupture is one of the most common injuries in playing rugby. The aim of this study was to assess the effect of a custom-designed training program on changes in dynamic knee valgus angle (DKV) and Reactive Strength Index (RSI), which are the main risk factors, in a group of female Rugby-7 players. **Methods:** A total of 16 professional Rugby-7 players completed an 8-week KAT program intervention, which was incorporated twice a week throughout this time. In both the pre- and post-tests, dynamic knee valgus was assessed during the drop jump (DJ) test using the frontal plane projection angle (FPPA) method. The jumps were analyzed using Dartfish 2024 software. **Results:** The post-tests revealed an increase in RSI values (*p =* 0.0496; SD = 1.25 ± 0.44 vs. 1.40 ± 0.35) and a reduction in valgus of the left knee joint (*p =* 0.01; SD = 9.08 ± 11.86 vs.0.00 ± 7.42). The correlation between RSI and the valgus angle produced inconclusive results (rs = −0.69; *p* < 0.01; rs = −0.35; *p =* 0.25; rs = −0.38; *p =* 0.2; rs = −0.2; *p =* 0.51). Cohen’s d = −0.37964. **Conclusions:** The training program proved effective in improving RSI scores and reducing the valgus angle of the left lower limb, which functioned as the supporting leg. These findings potential KAT implementation as a warm-up routine in professional women’s rugby clubs.

## 1. Introduction

Rugby is growing rapidly among female athletes globally, with a 11% increase in participation and 37% rise in registered players reported in 2023 [1]. In Poland, the seven-a-side variant (Rugby-7) dominates women’s competitions. However, this format’s high-intensity, multi-match tournaments elevate injury risks [2]. During the 2023/2024 season, 384 injuries were recorded in women’s Rugby-7, translating to 97.5 injuries/1000 play hours (95% CI: 88.3–107.6), with an average severity of 49.2 days lost (95% CI: 42.7–55.8) [3].

Modifiable factors that predispose individuals to knee join injuries include lack of proper range of motion [4], improper biomechanics during landing after jump, and inadequate postural control [5]. Those also contribute to dynamic knee valgus (DKV). This abnormal movement pattern combination of hip adduction and internal rotation, anterior translation and external rotation of the tibia, and eversion of the ankle joint [6] increases ACL strain, anterior knee pain often referred as a “runner’s knee”, patella lateralization, and accelerates chondromalacia [7,8,9]. Moreover, according to the research by Fuller and Taylor (2020), knee injuries, particularly ACL tears, were not listed among the most common injuries in male competitions [10] but disproportionately affect female players, accounting for 21.5% of total time lost from play compared to 10.9% [11].

The measurement of DKV is conducted while the athlete performs a movement task. One of the most used tests for this purpose is the drop vertical jump (DVJ) or drop jump (DJ) [12]. This test is not only utilized for injury risk assessment but also for evaluating motor potential. The drop vertical jump (DVJ) test is widely used to evaluate DKV and neuromuscular control. It demonstrates high inter-rater reliability (κ = 0.92; 95% CI: 0.829–0.969) and concurrently measures the Reactive Strength Index (RSI), a marker of stretch-shortening cycle efficiency calculated as jump height divided by ground contact time.

The achieved jump height serves as an indicator of an athlete’s explosive power [13] and demonstrates a high level of inter-rater reliability (κ = 0.92; 95% CI = 0.829–0.969; *p* < 0.05). Additionally, it has a sensitivity of 95% and a specificity of 46% [14]. The Reactive Strength Index (RSI) is one of the methods used to assess the function of the stretch-shortening cycle [15]. Plyometric exercises are particularly well-suited for the purpose of determining RSI, as they enable the generation of high force in a very short time [13]. As such, the Index is also used as an ACL tear predictor [16]. The literature describes three different formulas for calculating RSI [17], with the most accurate method being the calculation of jump height based on ground reaction forces, when a force plate is available [18].

While 3D motion analysis remains the gold standard for DKV assessment [19,20], 2D video analysis offers a practical alternative, making it feasible for clinical and team settings. It involves recording movement or a functional test using a digital camera positioned directly in front of the participant and then importing the footage into video analysis software (e.g., Dartfish, Quintic, Kinovea) for evaluation [21]. Using the appropriate technique, it is possible to determine the dynamic knee valgus angle. Studies comparing the 2D method to 3D analysis have shown high reliability (ICC = 0.95 to 0.99) and strong correlation of results (ICC: 0.96; 95% CI: 0.82–0.98; ICC: 0.94; 95% CI: 0.90–0.96) [22]. This allows the results of functional tests to be easily objectified and the identification of abnormal movement patterns [23].

Since the available literature has shown that knee injuries in women’s competitions occur frequently and are associated with long recovery periods, this study focused on prevention in this area [24]. Due to the high injury rate in rugby, implementing preventative measures is essential. In sports such as basketball, soccer, and handball, differences in injury incidence between genders have been documented [25,26,27]; however, most studies on rugby injuries focus solely on men’s competitions, making it necessary to conduct research specifically on female athletes.

There are already many preventative training programs [28,29]. One of them is the FIFA 11+ program, which was specifically designed to combat the occurrence of non-contact injuries. It is intended for both male and female players over the age of 14. The program should be conducted as a warm-up before the main part of a training session and to be effective, it must be performed at least twice a week [30]. Activate is a warm-up exercise program designed to be used three times per week prior to rugby training and matches [29]. Moreover, the effectiveness of preventative programs also depends on the correct execution of exercises, through neuromuscular training [31]. However, none of these programs specifically target the joints most vulnerable to injury in rugby–the knee joints—despite the existing literature highlighting the necessity of such an intervention. This gap underscores the need for a tailored intervention and led to the development of the Knee Armor Training (KAT) protocol.

The aim of this study was to evaluate the efficacy of the Knee Armor Training (KAT) protocol–an 8-week program targeting DKV and RSI–in improving knee stability among female Rugby-7 athletes, and to check if KAT will significantly reduce DKV angles and increase RSI values by enhancing neuromuscular control and plyometric capacity.

## 2. Material and Methods

The study group consisted of female rugby players. The selection was intentional, with inclusion criteria requiring at least two years of experience in Rugby-7, no current lower limb injuries, and an age range of 18 to 35 years. A total of 24 athletes were recruited; however, due to unforeseen circumstances and player transfers, 16 participants completed this study.

The absence of a control group in this study was a deliberate methodological choice aligned with the research’s pragmatic focus on real-world applicability. The intervention targeted a homogeneous cohort of elite female rugby players from a single professional club (Legia Warszawa), where logistical constraints (e.g., limited roster size, synchronized training schedules) made randomized group allocation unfeasible. By adopting a pre-post intervention design, this study prioritized assessing individual responsiveness to the training program within this specific athletic population, mirroring practical scenarios where entire teams adopt preventative protocols without parallel control groups. This approach is consistent with similar sports science studies evaluating training efficacy in elite cohorts.

The athletes underwent pre-tests, followed by an 8-week custom intervention based on the FIFA-11 and Activate protocols [31], and then post-tests. This study received approval from the local ethics committee in Poland (SKE 01-44/2022), and all participants provided written informed consent. The anthropometric characteristics of the athletes are presented in Table 1.

Both before the pre-tests and post-tests, a standardized, supervised warm-up was conducted. Next, bony landmarks were marked on the athletes’ bodies using a black marker, specifically the tibial tuberosity on both lower limbs, which served as a reference point for measuring knee valgus during recordings.

The athletes then performed the drop jump (DJ), which involved stepping off a 40 cm high wooden platform onto the center of a force plate positioned 40 cm away, landing on both feet, and immediately executing a maximal vertical jump. A JBA Staniak force plate (80 × 80 cm JBA Staniak, Warsaw, Poland) with MVJ6v0 software (JBA Staniak, Warsaw, Poland) was used to measure lower limb power and calculate the RSI (the ratio of jump height to ground contact time). The RSI measurements were obtained using the DJ with both feet simultaneously on the platform mode.

Jump height was determined using ground reaction force data recorded by the force platform, allowing for objective measurement. The Reactive Strength Index (RSI) was then calculated using the formula shown below [32,33]:

$$\mathrm{RSI}=\frac{jump\;heigh}{time\;of\;contact\;with\;the\;platform}$$

To dynamically assess knee valgus, the jumps were recorded using a smartphone (Samsung Galaxy S20FE, Samsung Group, Suwon, South Korea) in super slow-motion mode, positioned 220 cm from the platform. The recordings were analyzed to measure the dynamic knee valgus angle with an accuracy of 0.01° using the Dartfish Live software within the Dartfish 2024 (Dartfish, Fribourg, Switzerland) application (with software 11.3.1115.0).

Each athlete performed three drop jump trials. The trial in which the athlete achieved the highest jump was selected for measurement, and the moment of maximum knee flexion during landing was used to assess the knee valgus angle. Maximum knee flexion was defined as the video frame immediately before the knee joint began extending for the take-off phase.

Regarding outlier management, the protocol emphasized maximizing data integrity rather than exclusion. Participants performed three trial repetitions of the drop jump test to account for intra-athlete variability and mitigate measurement errors inherent in dynamic tasks. The selection of the best-performing trial (highest jump height) ensured the analysis reflected each athlete’s peak capacity, a common practice in performance testing to capture “game-ready” neuromuscular output. While explicit outlier criteria (e.g., ±3 SD) were not applied, the triplicate trial design inherently reduced the impact of anomalous single attempts without discarding participants-a critical consideration given the small sample size (n = 16 post-attrition). This strategy balanced methodological rigor with the practical reality of limited athlete availability in elite sports.

**Figure 1:**
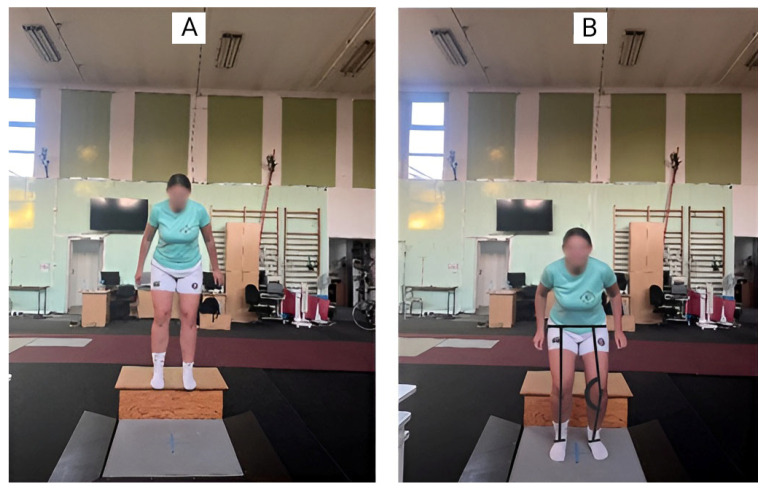
Start (**A**) and end position (**B**) in DJ test.

To determine the dynamic knee valgus angle, a straight line was drawn bisecting the thigh, ending between the lateral and medial epicondyles, and another line was drawn separating the lower leg, ending between the lateral and medial malleolus [22]. Based on this method, DKV was calculated using the FPPA method for both the right and left limbs. The DJ test is illustrated in Figure 1.

### 2.1. Intervention

After the first testing session, an intervention was introduced in the form of a custom-designed training program aimed at improving knee joint stability, balance, and muscle strength. It was incorporated into regular training sessions before the main part of practice. The program was applied twice a week, with each session lasting 45 min, for a total duration of eight weeks. The exercises performed by the athletes are presented in Table 2.

The KAT program was conducted by the team coach, who had been previously introduced it to and taught the program. The correctness and the fact of performing the exercises were checked by unannounced and randomly occurring visits of researchers during training sessions.

### 2.2. Statistical Analysis

Statistical analysis was performed using the Statistica software (version 14, TIBCO Software Inc., Palo Alto, CA, USA, 2017). The normality of the variable distribution was assessed using the Shapiro–Wilk test. For all data, means, standard deviations, minimum, and maximum values were calculated. To compare data before and after the intervention, the Wilcoxon signed-rank test and the paired *t*-test were applied. A significance level of *p* < 0.05 was used for all tests and Cohen’s |d| = 0.2 (small effect), 0.5 (moderate), and 0.8 (large) were used to interpret strength of the comparison.

To examine the relationship between RSI and knee valgus angle, Spearman’s rank correlation analysis was conducted. The correlation strength was interpreted as follows: trivial (r = 0.0–0.1), small (r = 0.1–0.3), moderate (r = 0.3–0.5), strong (r = 0.5–0.7), very strong (r = 0.7–0.9), and nearly perfect (r = 0.9–1) [16].

## 3. Results

The results of RSI and knee valgus angles in the pre- and post-tests are presented in Table 3. Positive angle values indicate valgus, while negative values indicate varus.

**Figure 2:**
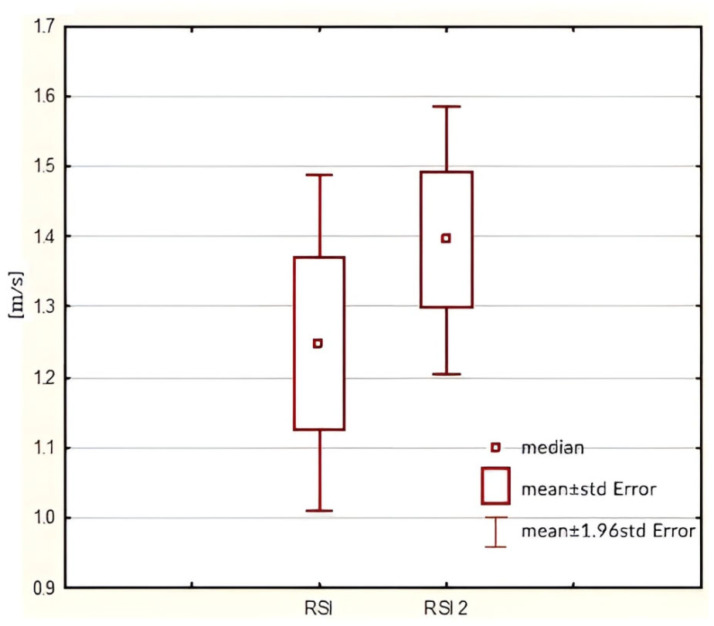
RSI values in pre- (RSI) and post-tests (RSI2) graph.

The statistical analysis revealed a statistically significant increase in RSI values in the post-tests compared to the pre-tests (*p =* 0.0496). Detailed results of this analysis are presented in Figure 2.

**Figure 3:**
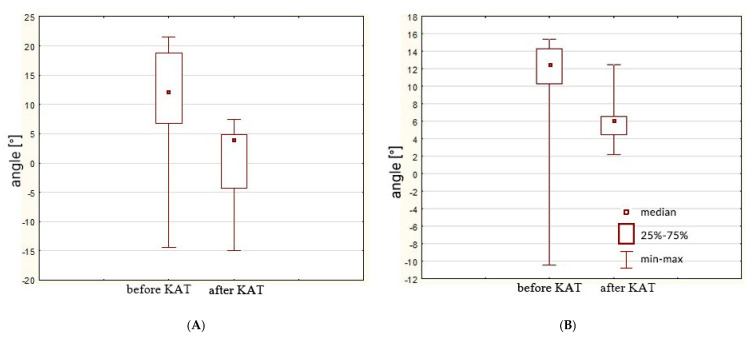
Changes in knee valgus angle values [°] for the left (**A**) and right (**B**) lower limbs after KAT training.

For knee valgus angles, the left knee reduction (9.08° to 0.00°) demonstrated a large effect size (d = 0.92), aligning with benchmarks for biomechanically relevant changes in ACL loading. However, the right knee’s non-significant trend (*p* = 0.06; d = 0.61) underscores the importance of reporting CIs: the 95% CI [0.24, 6.72] indicates plausible meaningful improvements that this study may have been underpowered to detect conclusively. The results are presented in Figure 3.

Additionally, RSI values in the pre-tests showed a strong negative correlation with the valgus angle of the right lower limb (rs = −0.69; *p*< 0.01), while no significant correlation was found with the angle of the left limb (rs = −0.35; *p=* 0.25). RSI values in the post-tests did not show significant correlations with the valgus angle of either the right (rs = −0.38; *p =* 0.2) or left lower limb (rs = −0.2; *p =* 0.51).

## 4. Discussion

Given the high injury rate in this sport, there are significant considerations regarding the costs of surgical treatment and rehabilitation, the potential for missing entire seasons, and long-term consequences such as early degenerative changes in the knee joints or an increased risk of subsequent injuries [34,35].

The main observation from this study is the positive impact of the custom training program on dynamic parameters defining knee joint stability in professional female rugby players. This study is the first in female rugby players to examine the hypothesis that dynamic knee valgus (DKV), reactive strength index (RSI), and lower limb asymmetry—as recognized predictors of lower limb injury—can be ameliorated by a targeted intervention combining biomechanical assessment and a KAT protocol. This program, developed by the authors, integrates neuromuscular control exercises and asymmetry correction strategies with the goal of establishing a new framework for injury prevention in this high-risk group.

The effectiveness of the FIFA 11+ program was demonstrated in a study conducted on young female soccer players in Norway, where it reduced the incidence of injuries by up to 50% [36]. An attempt to implement the FIFA 11+ program in basketball achieved partial success. While the overall incidence of lower limb injuries was significantly lower among young basketball players, no difference in knee injury frequency was observed between the control and experimental groups [37]. Surprisingly, in American football players, the FIFA 11+ program did not help reduce lower limb injuries, but those who did a regular warm-up had fewer injuries per 1000h of play [38]. It has been proven that after properly completing the FIFA 11+ program, the following aspects improve: neuromuscular control, static and dynamic balance, agility, lower limb muscle strength, muscle imbalance, and jump performance [39,40,41]. Additionally, exercises incorporating unstable surfaces, such as a sensorimotor cushion, have been proven to be effective and essential in designing injury prevention training programs [42,43]. Instability, detected by the muscle-tendon unit, triggers a response from the central nervous system to recognize and correct the movement to a proper pattern [43]. Additionally, when designing training programs, it is important to incorporate balance exercises that include elements relevant to in-game movements [44]. In rugby, players engage in short but highly intense anaerobic activities such as sprints and explosive force bursts to evade opponents [45]. Sudden changes in direction require players to generate maximum power in the shortest possible time. Various aspects of the game, such as tackling and scrum, demand significant muscle strength development [46]. As a result, players must develop motor skills such as aerobic and anaerobic endurance, speed, strength, jumping ability, and agility [47].

Since jump training has been shown to reduce dynamic knee valgus and lower the incidence of ACL injuries, elements of box jumps were incorporated into the program, with the primary focus on controlling movement patterns [48,49]. Understanding the injury mechanism, learning how to correct improper limb positioning, and reinforcing proper posture are key components of injury prevention programs [28].

The custom training program, inspired by the FIFA 11+ and Activate protocols, successfully increased the RSI in this study group. Improved post-test results indicate enhanced neuromuscular control among the athletes, which is crucial for the optimal distribution of forces on the muscle–tendon unit during rugby-specific movements such as sudden changes in direction or landing on one leg [50]. Sprints and jumps require the efficient functioning of the stretch-shortening cycle [15]. It is defined as the sequence of muscle activity in natural human movements, where the first phase is an eccentric contraction followed by a concentric contraction. Its purpose is to enhance force generation compared to a purely concentric contraction that does not involve prior muscle stretching. Additionally, this mechanism helps conserve energy by reducing the metabolic cost of movement [51]. In clinical and club settings, assessing RSI in rugby players is beneficial not only for evaluating plyometric performance or tracking training progress. This index also serves as a valuable tool for assessing the risk of ACL injuries [15]. It is recognized that the lower the RSI value, the weaker the function of the stretch-shortening cycle. This, in turn, affects movement efficiency and increases the risk of injury in athletes. It is also important to note that there are challenges in interpreting RSI values in rugby players. These challenges arise from the use of different tests to assess RSI, variations in calculation methods, and, most importantly, the diversity of sports disciplines. In his study, Flanagan (2016) established RSI threshold values for the drop jump test, using a calculation method that takes into account jump height and ground contact time [52]. However, the author emphasizes that the data used for these benchmarks were derived from multiple sports disciplines, each with different demands for explosive force generation. When comparing the results of female rugby players, it appears that their average reactive strength index (RSI = 14) is surprisingly low, placing them in the weakest performance group (Figure 3). Interpreting these results suggests that the athletes are not adequately prepared even for moderate-intensity exercises. Their training should focus on the most basic plyometric drills and increasing lower limb muscle strength. The impact of another custom training program, “KneeRugbyWoman”, on RSI values was also studied in amateur rugby clubs in the Czech Republic [15]. The training program included strength, balance, and plyometric components. After 12 weeks, the athletes were re-evaluated, successfully achieving an increase in RSI values. However, comparing the values between Czech and Polish athletes is nearly impossible. A different test than the drop jump was used to determine the RSI, resulting in significantly lower average values for the Czech rugby players (RSI = 0.86). For this reason, Flanagan et al. (2016) suggested that sports clubs should independently create their own datasets for their athletes. This would allow RSI to serve as an individual performance indicator, reflecting progress, neuromuscular control, and fatigue. Additionally, it could be used as a recovery metric, indicating an athlete’s readiness to return to training after an injury [52,53].

Frontal plane projection angle (FPPA) is a technique used to determine the knee valgus angle in the frontal plane. The main challenge with this method appears to be the lack of a standardized approach for defining the lines that form this angle. It is determined by connecting two straight lines running through the thigh and lower leg; however, variations in research arise due to different starting and ending points for these segments.

In some studies, the first line extends from the anterior superior iliac spine to the center of the knee, which is described as the midpoint between the lateral and medial femoral condyles. The second line runs from this knee point to the center of the ankle, defined as the midpoint between the lateral and medial malleoli of the tibia [22,54,55,56].

The scientific literature also describes alternative methods for determining the angle between the thigh and lower leg. In these approaches, a straight line is drawn bisecting the thigh, ending at the intersection of the femoral epicondyles. From this point, another straight line is drawn, bisecting the lateral and medial malleoli of the tibia [23].

Unfortunately, no studies have yet been published comparing the accuracy or reliability of these different methods for measuring knee valgus angle. This may present a limitation when comparing publications that use different techniques. Nevertheless, FPPA allows for the objectification of functional test results, making it possible to compare a patient’s outcomes before and after a given therapy or intervention. Additionally, various studies describe different methodologies for the DJ test, with variations in drop height, landing distance, and specific execution instructions [57,58].

However, no studies have compared different drop heights and their impact on DKV. Due to the lack of existing publications measuring FPPA in female rugby players during the DJ test, it was not possible to reference a specific procedure. Therefore, a custom methodology was applied. Observing its impact on dynamic knee valgus, the training program was statistically successful. The mean valgus angles of both lower limbs decreased following the custom intervention. It is worth noting that a statistically significant difference between pre- and post-tests was observed in the left lower limb, which served as the supporting leg. In summary, the training proved to be an effective tool in reducing the average dynamic knee valgus angle, which in turn indicates a reduced risk of knee joint injuries [28].

In 2010, Herrington published reference values for dynamic knee valgus angles in physically active men and women, with the inclusion criterion being at least three hours of physical activity per week. Comparing these data shows that before the intervention, female rugby players exhibited higher valgus angle values than the women in Herrington’s study (L = 8.2° vs. 9.08°, *p* = 9.9° vs. 10.0°) [55]. Additionally, nearly half of the Polish athletes (7 out of 16) exceeded the reference values proposed by Herrington. In his study, he suggests that achieving more than 7–13° in the drop jump is significantly associated with an increased risk of ACL and patellofemoral joint injuries. Thanks to the applied intervention, every athlete remained within the proposed normative range (max = 12.5°), which can be considered a significant success demonstrating the effectiveness of the custom training program. Since RSI values and dynamic knee valgus angles are commonly used in research to assess injury risk and can be measured using the same test, it was decided to examine whether there is a correlation between them [59,60]. Due to the association of RSI with better neuromuscular control, strength, and movement efficiency, it was hypothesized that individuals with a higher RSI would exhibit a better movement pattern, meaning a smaller dynamic knee valgus angle [61]. Unfortunately, the results are inconclusive and insufficient to confirm this hypothesis. Only the pre-test data showed the expected outcome for the right lower limb, with a strong negative correlation (rs = −0.69; *p* < 0.05). The current literature does not provide any studies examining the relationship between these two parameters, making this an important area for future research. Nevertheless, the KAT custom training program contributed to reducing modifiable risk factors for knee joint injuries.

### 4.1. Limitations of the Study

The current research had some limitations. To confirm its effectiveness in lowering injury rates, long-term observation of athletes over multiple seasons would be necessary. Unfortunately, this extends beyond the scope and objectives of this study but leaves room for future research in this direction. This study has several limitations that should be acknowledged. The most significant is the small sample size, which may reduce the reliability of the findings and create challenges in interpreting the results. The small group of participants in this study is the result of the small overall population of professional female rugby players, whose number in Poland—despite the growing interest in this discipline—oscillates around 150–200 women (2–3% of all registered rugby players in Poland). It was therefore decided to maintain the homogeneity of the group (research conducted on one team) to exclude additional variables. However, this necessitates extending the research to other rugby teams or even other disciplines in order to be able to determine with greater certainty the effect of using KAT. Therefore, future researchers are encouraged to gather a larger study group to improve the reliability of the findings and assess the long-term effects of the custom training program.

There was no real possibility of blinding this study (participants, coaches or analyzers) in this particular research project. This is typical of many intervention studies in sports, where both participants and researchers are aware of the intervention and its purpose.

Another limitation is the focus solely on non-contact knee injuries. In rugby, a significant proportion of injuries also result from contact events, such as tackles [24]. Injuries in rugby affect not only the knees but also the shoulders and the head and facial area. Since this was one of the first studies on the implementation of a preventative training program for women in rugby, it is suggested that future research could modify the program by incorporating additional exercises or elements aimed at preventing these types of injuries.

### 4.2. Practical Application

The findings of this study suggest that implementing a structured training program focused on neuromuscular control, proprioception, and explosive strength can enhance performance and reduce injury risks for female Rugby-7 players. The exercises targeting knee stability, balance, and muscle strength were shown to positively influence movement patterns and injury prevention. Integrating this program into standard training routines may improve knee alignment, reactive strength, and overall biomechanics, which should lower the risk of ACL injuries and lower limb dysfunctions. Given the high injury rate in rugby, particularly non-contact knee injuries, incorporating these exercises into warm-up sessions could be a valuable strategy for long-term injury prevention and athletic development.

## 5. Conclusions

The assessment of dynamic knee valgus angle and RSI helps identify athletes at an increased risk of knee joint injuries. This study demonstrates how a simple diagnostic test can enable clinicians and coaches to evaluate both parameters effectively.

1. Professional female rugby players often display improper movement patterns, weak neuromuscular control, and insufficient explosive strength. These factors increase the risk of knee injuries, including ACL tears, highlighting the need for a targeted training program to address these weaknesses and reduce injury risk.
2. The custom training program KAT should be incorporated into regular rugby training, as it is safe to perform and has been shown to improve key dynamic stability measures of the knee joint when applied consistently.

## Figures and Tables

**Table 1:** Group anthropometric characteristics.

	n	Age [Years]	Body Weight [kg]	Body Height [cm]
players	16	23.0 ± 4.33	69.83 ± 11.84	168.0 ± 5.85

**Table 2:** Exercises of the Knee Armor Training (KAT) protocol.

Exercise Name	Description	Time/Repetitions
Balance Pads and Ball Throwing	Athletes stand opposite each other on balance pads. Step onto the pad, catch the ball ⟶ throw it back ⟶ step down. Three athlete switches.	15 reps per set
Balance Pads–Lunges	Lunge onto the balance pad, push off with the front leg, and return.	Each leg ×45/alternating
One-Leg Clock	Standing on one leg, reach with the lifted leg as far as possible along the lines on the floor (coach signals the ‘hour’ direction).	1 min per leg ×3
One-Leg Pushing	Athletes stand on one leg and push against each other, trying to off-balance partner.	1 min per leg ×3
Box Jump Landing	Jump off a 30 cm box and land with a loud stomp, holding a half-squat.	15 reps
Lateral Step	Step laterally in the direction indicated by a partner or coach (3 steps in the signaled direction, then quick return).	1 min
Star Jumps	Jump with both feet in 8 directions as indicated by the partner or coach, returning to the start position between jumps.	30 s–1 min
Lateral Jumps with Weight	Wide lateral jumps (alternating L and R), landing on one leg while holding a heavy weight (10/15 kg) in front.	45 s
Skip A+	Skip A with a two-foot stomp on signal.	30 s
Crab Walk	Lateral step in a low squat position, resistance band below knees.	1 min per side
Side Plank	In a side plank, raise and lower the hip then switch sides.	45 s per side
Copenhagen plank	In a side plank, place the upper leg on an elevated surface (chair/bench). The lower leg is lifted towards the upper leg and lowered.	45 s per side
Hamstring Lean	In a kneeling position, lower the torso forward while keeping the calves stabilized (Nordic curl).	30 s
Single Leg RDL	Standing on one leg (slightly bent), hold a kettlebell in the opposite hand. Lean forward, touching the kettlebell outside the support leg, with the airborne leg parallel to the body.	45 s per side
Lower Limb Stabilization with Assistance	Partner exercise–one person in long sitting position, the assistant supports the heel 10 cm above the ground. The athlete presses the heel against the assistant’s hand (isometric) for 2–3 s in 8 different directions (down, side, up, up and inside, etc.).	2–3 min per limb, switching athletes

**Table 3:** Descriptive statistics (mean, standard deviation, minimum, maximum) for RSI and knee valgus angles before and after the intervention.

Variable	Test	Median	SD	Minimum	Maximum
RSI [a.u.]	Pre-test	1.25	0.44	0.73	2.25
RSI [a.u.]	Post-test	1.40	0.35	0.87	1.97
FPPA R [°]	Pre-test	10	7.46	−10.38	15.4
FPPA R [°]	Post-test	6.52	3.13	2.20	12.48
FPPA L [°]	Pre-test	9.08	11.63	−14.42	21.53
FPPA L [°]	Post-test	0	7.42	−14.89	7.53

Legend: RSI—Reactive Strength Index; FPPA—frontal plane projection Angle; R—right lower limb; L—left lower limb. Positive angle values indicate valgus, while negative values indicate varus.

## Data Availability

The original contributions presented in this study are included in the article. Further inquiries can be directed to the corresponding author(s).
